# Nitriding Effect on the Tribological Performance of CrN-, AlTiN-, and CrN/AlTiN-Coated DIN 1.2367 Hot Work Tool Steel

**DOI:** 10.3390/ma16072804

**Published:** 2023-03-31

**Authors:** Gülşah Aktaş Çelik, Şaban Hakan Atapek, Şeyda Polat, Aleksei Obrosov, Sabine Weiß

**Affiliations:** 1 Laboratory of High Temperature Materials, Department of Metallurgical and Materials Engineering, Kocaeli University, İzmit 41001, Türkiye; 2 Department of Physical Metallurgy and Materials Technology, Brandenburg University of Technology Cottbus—Senftenberg, 03046 Cottbus, Germany

**Keywords:** DIN 1.2367, PVD coating, wear, characterization

## Abstract

In this study, heat-treated and multisurface engineered DIN 1.2367 tool steel was subjected to room and elevated temperature wear tests, and the effect of nitriding on its tribological behavior was investigated. CrN, AlTiN, and CrN/AlTiN coatings with a total thickness of 2 µm were obtained by arc cathodic physical vapor deposition on conventional heat-treated and gas-nitrided steels. The white layer formed during nitriding was removed, and a diffusion layer (100 µm) was achieved in the cross section of the steel having a tempered martensitic matrix. The highest surface hardness was attained with an integral coating (CrN/AlTiN), and surface hardness increased even more after nitriding due to the formation of a multicomponent ceramic layer on top of the diffusion layer. The room temperature wear tests performed against an alumina counterpart revealed that (i) CrN/AlTiN-coated steel had the highest friction coefficient of 0.26, which further increased to 0.33 by nitriding due to the increase in shear strength, and that (ii) with increasing surface hardness, the specific wear rates (W) of the heat-treated and coated steels could be ranked as follows: W_CrN/AlTiN_ < W_AlTiN_ < W_CrN_. The wear rates decreased when nitriding was carried out prior to coating. In order to simulate the aluminum extrusion conditions, hot wear behavior of the surfaces against AA6080 alloy at 450 °C was investigated. The hot wear tests revealed that (i) high friction coefficients were reached due to the adhesive characteristic of aluminum to the surfaces, (ii) the nitrided and CrN/AlTiN-coated sample exhibited the lowest wear rate among all studied surfaces, and (iii) the film damage on the worn surfaces mostly occurred in the form of droplet delamination.

## 1. Introduction

Steels are commonly used, among other things, in the fabrication of medical devices, equipment, and machine parts in various industries, where good mechanical and tribological properties are required [1,2]. Different treatments, such as physical, chemical, and thermal surface treatment procedures, can be used to enhance their surface properties [3]. All these methods have corresponding disadvantages. Nitriding does not provide sufficient resistance in highly aggressive environments or under significant mechanical loads, which limits the feasibility of this method despite the significant improvement in surface hardness and wear resistance compared to untreated steel [4]. PVD hard coatings also have significant hardness and wear resistance, but the adhesion strength of the deposited coatings is still a serious problem that necessarily requires pretreatment of the parts [5]. An important factor is the difference between the mechanical properties of the applied coating and the ones of the substrate material due to which adhesion can be strongly reduced [6]. Interfacial adhesion can be significantly improved by changing the mechanical parameters of the material surface before coating deposition is performed [7] by nitriding or heat treatment of the surface [8]. The combination of nitriding and subsequent coating is called duplex surface treatment technology, which can be used for different applications depending on the specific requirements [9,10].

Combinations of coatings and surface modification have been presented by numerous authors [4,11,12]. Due to their remarkable mix of characteristics, binary, and ternary transition, metal nitrides are frequently employed as single-layer coatings for industrial purpose to enhance the efficiency of tools. Compared to other nitride coatings, chromium nitride coatings demonstrate superior oxidation resistance [13]. Due to their high temperature stability, high hardness, superior toughness, good wear resistance, and lower friction coefficient than TiN, chromium nitride has received much attention in a variety of applications [14]. Additionally, CrN coatings provide a low thermal conductivity coefficient [15], which is crucial for reducing plastic deformation of coatings applied for protection on forging dies [16]. The hardness of the CrAlN coating system is considerably increased by adding Al to the cubic CrN structure [17]. Numerous studies have demonstrated the improved abrasion, corrosion, and oxidation resistance as well as the tribological properties of CrAlN coatings [18,19,20]. An addition of Al into CrN leads to a further slight decrease in thermal conductivity of CrAlN coatings [15]. Siddiqui et al. [11] reported a 53% improvement in the adhesion strength of CrN coatings deposited on duplex-treated tool steel. Alkan et al. [21] described that plasma nitriding before coating significantly improves corrosion and tribo-corrosion resistance of PVD CrN, TiN, and AlTiN coatings.

The parameters of nitriding are also important and should be considered. With knowledge of the mechanisms of nitriding, it is possible to selectively set very different surface conditions in components and tools [22]. An increase in ion current density can strongly improve nitriding efficiency, as demonstrated by Zhang et al. [23]. Combining nitriding pretreatment with coating deposition increases adhesion and wear resistance of CrAlN coating. Some groups have already made improvements to X38CrMoV5-3 (DIN 1.2367) steel used to produce forging tools [16,24]. Pashke et al. [24] discovered that by choosing the right parameters of plasma nitriding, crack behavior of hot-forming tool steel can be effectively modified. Smolik et al. [16] evaluated the effects of a nitrided layer/PVD coating composite structure on the service life of forging tools made of DIN 1.2367 steel. They reported that high thermal fatigue resistance and wear resistance were achieved.

Despite many papers on duplex-treated tool steels, there are not enough systematic studies on the tribological behavior of X38CrMoV5-3 (DIN 1.2367) steel at room temperature and especially at elevated temperatures. The aim of the present study was to develop a heat- and wear-resistant duplex surface treatment to improve the lifetime of tools.

## 2. Materials and Methods

In this study, the hot work tool steel DIN 1.2367 (0.38C, 0.30Mn, 0.30Si, 5.00Cr, 3.00Mo, 0.60V, wt.%) was used. The samples with a size of 20 × 20 × 20 mm were mirror-polished with 3 µm diamond paste to obtain a roughness parameter (R_a_) of 0.02 µm. The polished samples were washed in an alkaline solution, rinsed in distilled water, and dried in warm air before heat treatment and deposition. During heat treatment, the samples were preheated twice: (i) 600–650 °C for 60 min. and (ii) 800–850 °C for 60 min. Subsequently, the samples were austenitized at 1030 °C for 30 min. and quenched in air. The tempering process was carried out in three steps by annealing at (i) 585 °C for 120 min., (ii) 560 °C for 120 min., and (iii) 560 °C for 120 min. The heat-treated samples were gas-nitrided at 585 °C for 6 hours and cooled in air atmosphere at 1.1 bar prior to the deposition to build up functionally graded surfaces in terms of hardness. As a result of gas nitriding, it is inevitable that a brittle white layer forms on top of the substrate and a diffusion layer responsible for the load-bearing capacity forms underneath. However, this brittle white layer must be removed by polishing to ensure adhesion of the deposited material. The cathodic arc physical vapor deposition (CAPVD) process was used for the deposition of hard ceramic monolayer coatings (CrN and AlTiN) and bilayer coatings (CrN/AlTiN). The parameters used for the coatings are listed in Table 1. The average substrate temperature during the coating process was 400 ± 50 °C. For the CrN/AlTiN coating, initially a CrN coating (1 μm) and then an AlTiN layer (1 μm) were deposited to obtain a total coating thickness of 2 μm. The thickness measurements showed that the desired coating thicknesses were achieved. The samples were designated according to their heat treatment and coating processes for easy identification (Table 2).

In order to reveal the microstructural features of the steel studied, a standard metallographic procedure was applied, and the polished cross sections were etched with a 3% Nital solution. The microstructures as well as the thickness of the coatings were examined with a scanning electron microscope (SEM, Jeol JSM 6060, Akishima, Japan and Zeiss Evo MA15, Abingdon, UK) equipped with an energy-dispersive spectrometer (EDS, IXRF, Austin, TX, USA).

**Figure 1:**
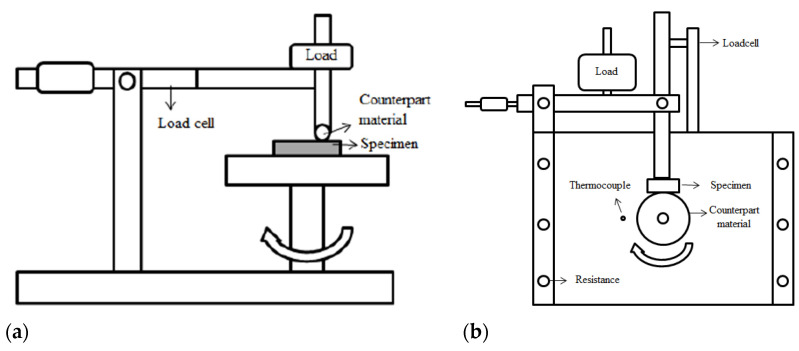
Configurations of (**a**) ball-on-disc and (**b**) block-on-cylinder types of tribometers used for RT and HT wear tests, respectively.

The mechanical properties of the heat-treated and coated samples were determined both by hardness measurements and tribological tests at room (RT) and high (HT) temperatures. For the hardness measurements, a microhardness tester (Emcotest Durascan 70, Salzburg, Austria) was used with 0.01 kgf, and both average values and standard deviations were calculated after 10 measurements. RT tribological tests were carried out using a commercially available ball-on-disc type of tribometer (Nanovea M/NI/1-E, Madrid, Spain) with an Al_2_O_3_ ball (diameter of 5 mm) as a counterpart. During the tests, the rotation speed, normal load, and total sliding distance were adjusted as 0.08 m/s (150 rpm), 20 N, and 150 m, respectively. A setup with a block-on-cylinder configuration mounted inside a heating chamber was used for HT tribology tests at 450 °C to simulate aluminum extrusion conditions [25,26,27]. A cylindrical billet of A6080 (ϕ 100 mm × 30 mm) served as a counterpart using a normal force of 70 N, a sliding speed of 0.27 m/s (53 rpm), and a total sliding distance of 2000 m. The configurations for the tribology tests are shown schematically in Figure 1. The tribological data were evaluated based on variation in the coefficient of friction (COF) and the specific wear rate (W) and were supplemented by examination of the worn surfaces using SEM and optical profilometer (Nanovea PS50, Spain). The Archard equation was used to calculate the specific wear rate by dividing the volume loss (mm^3^) determined by profilometric measurements by the selected load (N) and the sliding distance (m).

## 3. Results and Discussion

### 3.1. Hardness Profile and Microstructural Features

**Figure 2:**
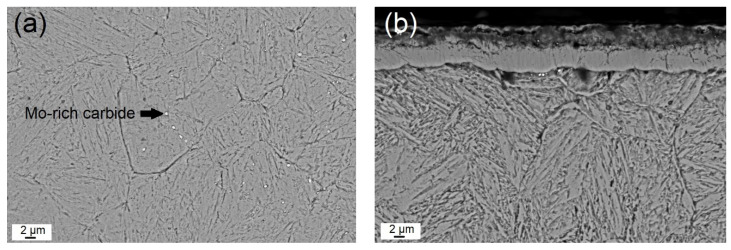
SEM images of the microstructure of the (**a**) heat-treated and (**b**) nitrided steel.

**Figure 3:**
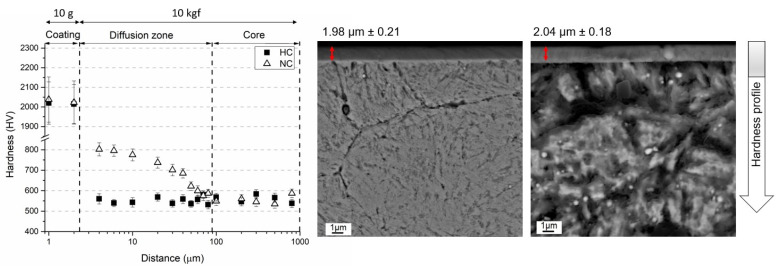
Hardness profile and coating structure of HC and NC.

**Figure 4:**
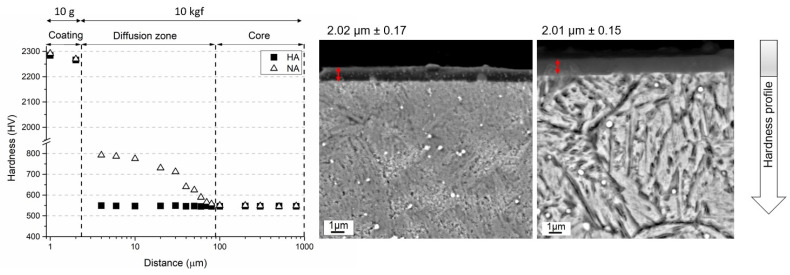
Hardness profile and coating structure of HA and NA.

**Figure 5:**
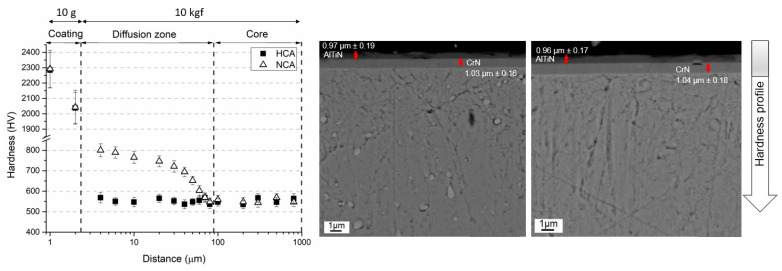
Hardness profile and coating structure of HCA and NCA.

The SEM image given in Figure 2a shows the general microstructure of the heat-treated steel. The microstructure consists of dispersed globular Mo-rich carbides embedded in a tempered martensitic matrix with a hardness of 538 ± 11 HV_10_. In Figure 2b, two distinct regions can be identified in the cross section of gas-nitrided steel: (i) a white layer known as the compound layer (γ’ and ε nitrides) with a thickness of ~10 µm and (ii) a diffusion layer (~100 µm) supersaturated with nitrogen underneath the compound layer with needlelike nitrides at the grain boundaries. As can be seen in Figure 2b, the surface layer is a typical white layer that has a strong tendency to adhere to the diffusion layer, although it was peeled off the surface due to the metallographic preparation with hard SiC particles. The hardness of the compound layer reached almost 1013 ± 28 HV_10_, while that of the diffusion layer below the interface with the compound layer was 798 ± 21 HV_10_. The hardness of the second layer gradually decreased towards the steel core as the solid solubility limit increased. The change in hardness with diffusion depth from the surface to the center can be seen in Figure 3, Figure 4 and Figure 5. The profiles indicate that the gas-nitriding process is successfully performed throughout the diffusion depth, similar to previous studies [27,28,29]. Furthermore, in these figures the microstructures of the coatings deposited on the steel substrate as well as their hardness profiles are shown. The coatings have continuous film layers of ~2 µm thickness that adhere well to the substrate materials and are free of pores, microcracks, and inhomogeneities. The CrN/AlTiN coating exhibits similar characteristics for both interfaces substrate/CrN and CrN/AlTiN. The cross section images also show that the white layer formed by nitriding is removed from the surface and that a hard ceramic-based deposition is achieved on the diffusion layer. Regardless of the coating process, equivalent hardness values are achieved in the diffusion layer of the substrate. So, there is a significant increase in hardness from the surface to the core of the substrate. To determine the chemical composition of the coatings, several EDS analyses were carried out. The results indicate that the CrN monolayer consists of 51% Cr and 49% N (at.%) and that the AlTiN monolayer includes 34% Al, 18% Ti, and 48% N (at-%). A similar chemical composition was determined in the CrN/AlTiN layer. The evaluation of the surface and subsurface hardness profiles shows that a hard coating of about 2018 ± 108 HV_0.01_ forms on the surface of the CrN-coated tool steel. The average hardness of the same layer deposited on the diffusion layer is 2032 ± 97 HV_0.01_. The hardness value of the AlTiN coating is higher than that of CrN. While the hardness value of the coating on the heat-treated steel surface is on average 2234 ± 127 HV_0.01_, this value can reach up to 2298 ± 151 HV_0.01_ for nitrided steel. It is well known that in PVD coatings, depending on the process conditions, various nano- to micro-sized defects can occur (nodular defects, pinholes, pores, and other coating discontinuities) that cause variations in hardness [30]. The large standard deviations of the hardness values indicate the presence of such defects even at a very low load (10 g). When determining the actual surface hardness of the single-coated multicoated samples, the hardness of both the substrate material and that of each individual coating is decisive. However, valid data can be obtained by means of Vickers hardness measurements that do not exceed 1/10 of the coating depth [31]. The surface hardness values of the heat-treated and the multisurface engineered steels are presented in Table 3. The comparison of the hardness values of the coating after a heat treatment resulting in a tempered martensitic matrix shows that the AlTiN coating has a higher hardness (1869 ± 90 HV_0.01_) than the CrN (1670 ± 85 HV_0.01_) coating and that the integral coating has the highest value (1987 ± 87 HV_0.01_). For coatings on the diffusion layer obtained by nitriding, a significant increase in all surface hardness values was observed. With nitriding and integral coating, the highest hardness was achieved with a value of 2692 ± 108 HV_0.01_.

### 3.2. Evaluation of the Tribology Test Results at RT

**Figure 6:**
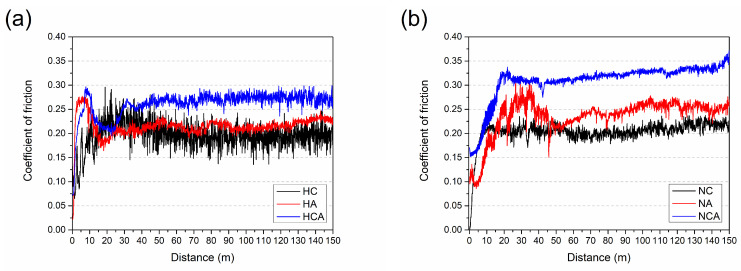
Friction coefficients as a function of distance obtained in RT wear tests for (**a**) heat-treated and coated and (**b**) nitrided and coated samples.

The coefficients of friction (COF) obtained after dry friction tests are given in Figure 6. The graphs mainly include run-in stage, transition stage, and steady-state stage. Considering the total test distance, there is a run-in stage in which the coefficient of friction increases linearly over the first 5 m of the HC, HA, and HCA surfaces (Figure 6a). However, when NC, NA, and NCA surfaces interact with alumina, this stage shifts to relatively large distances of up to 20 m (Figure 6b). Friction and wear changes that occur during the run-in stage are related to the changes in surface roughness, surface composition, microstructure, and third-body distribution depending on the tribosystem [32,33,34]. The mean values of surface roughness (R_a_) were determined for the heat-treated and coated samples and are 0.031 ± 0.006 µm, 0.033 ± 0.007 µm, and 0.030 ± 0.006 µm for HC, HA and HCA, respectively. These results indicate that the effect of surface roughness on friction and wear in the run-in stage is negligible. The COF values for HC, HA, and HCA vary between 0.20 and 0.25 in the run-in stage (Figure 6a) and can be attributed to the ploughing effect due to asperities and deformation of asperities resulting in polishing and adhesion on the surface at the beginning of the contact [35]. Previous studies revealed the tendency of the CrN layer to be removed from the surface in the early stages of tribological interaction, in contrast to the relative stability of the AlTiN layer [36,37]. As a result, the tribological pairs change from a ceramic/ceramic to a metal/ceramic contact and can cause a change in the wear mechanism from a two-body to a three-body contact caused by chipping [37]. Although there is no significant change in the COF values for NC and NA surfaces, the COF value of the NCA coating is higher than 0.30 due to the influence of hard particles present in the contact zone (Figure 6b). As expected, the shifting of the run-in stage of the nitrided and coated surfaces to longer distances causes a shift in the transition stage as well. For heat-treated and coated surfaces, the transition stage is between 5 m and 20 m (Figure 6a) and shifts to approximately 25–40 m for nitrided and coated surfaces (Figure 6b). This can be attributed to the higher surface roughness values (R_a[NC]_: 0.030 ± 0.007 µm, R_a[NA]_: 0.038 ± 0.009 µm, R_a[NCA]_: 0.032 ± 0.007 µm) of the nitrided and coated samples. Considering the average COF values of all surfaces in the steady state, the effect of both the coating type and the nitriding process becomes clear. For CrN and AlTiN coatings on the surface of the heat-treated steel, average COF values are 0.22 and 0.20, respectively. The average COF value for the HCA material belonging to the group of coatings with two ceramic layers on the surface increases and rises to 0.26 (Figure 6a). The highest COF value for the HCA coating, having the highest hardness among the heat-treated and coated steels, can be attributed to an increase in frictional force resulting from the increase in shear strength, which is the more dominant factor compared to the decrease in contact area [37,38,39]. This trend is also observed for nitrided and coated steels. Compared to heat-treated and coated steels, the average COF values increase for nitrided and coated steels. The average COF values determined for NC, NA, and NCA are 0.21, 0.25 and 0.33, respectively.

**Figure 7:**
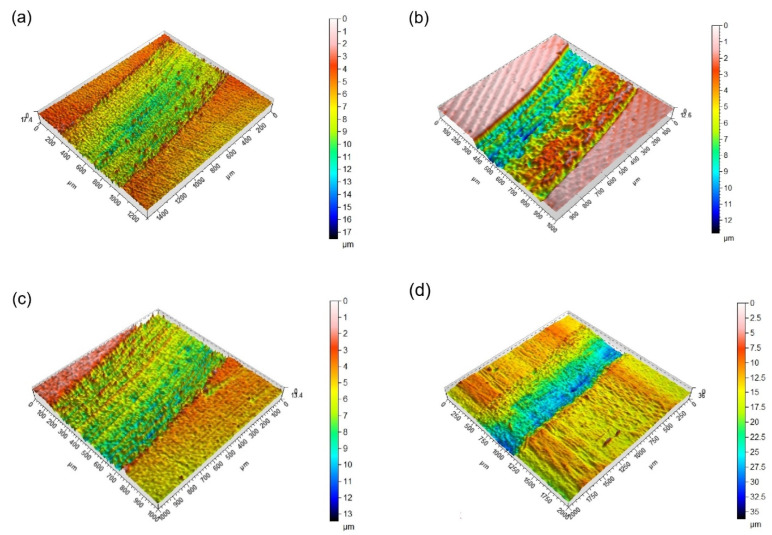

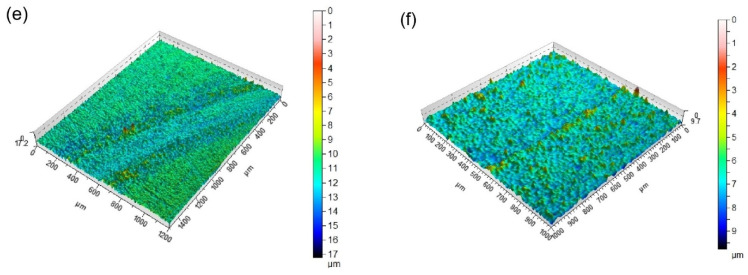
3D profilometer images of the wear tracks of all coated steels: (**a**) HC, (**b**) NC, (**c**) HA, (**d**) NA, (**e**) HCA, and (**f**) NCA.

Using 3D profilometry, wear traces on the worn surfaces are tracked, and depth profiles are created. Figure 7a shows the wear track on the surface of HC steel that interacted with the counterpart material under dry sliding conditions. The coating structure outside the wear track appears homogenous, as shown in the cross section studies. The width is measured to be about 800 µm. The track consists of both several grooves running parallel to the motion of the alumina ball and patches of the coating adhered to the track path (Figure 7a). The depth of the track (ΔZ) is measured to be about 19.2 µm, indicating that the CrN hard coating is completely removed, and the tribological pair consists of counterpart material and substrate during the steady-state stage. Similar track features are observed in the 3D track of NC; however, a narrower track of about 700 µm appears, and ΔZ is about 17.4 µm (Figure 7b). In this case, the CrN coating is also removed, but due to the harder surface, the contact area is reduced, resulting in a reduction in both track width and track depth. The high surface quality after coating can also be observed for HA, as shown in Figure 7c, and a harder surface compared to HC is reflected by a narrower (~550 µm) and flatter (~12 µm) wear track. Nitriding further reduces both width and depth of the track to ~500 µm and ~12 µm, respectively (Figure 7d). This is attributed to the fact that the contact area decreases with increasing surface hardness [40,41]. The tracks given in Figure 7e,f have a uniform appearance for the coatings, but a spalling effect due to adhesive wear during steady-state interaction between the counterpart material and the coating can be observed (Figure 7e). After nitriding, spallation is significantly less, and the coating remains on the surface (Figure 7f). Therefore, it is not meaningful to define track width and depth. The degree of wear during tribological interaction can be followed by the ratio of width to depth for heat-treated and multisurface engineered surfaces.

**Figure 8:**
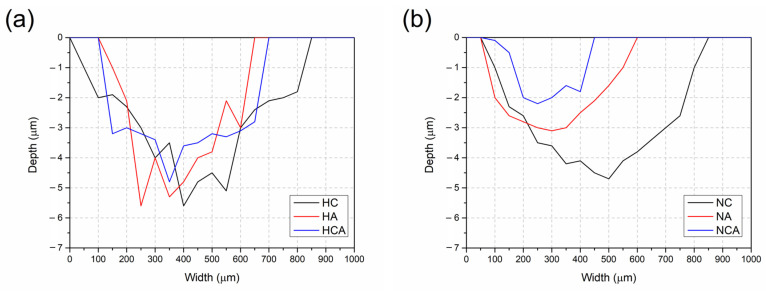
Track profiles of the surfaces after RT wear tests: (**a**) heat-treated and coated and (**b**) nitrided and coated samples.

In addition to the maximum track depth (ΔZ) on the wear surface, it is also important to consider the variation in depth and width of the track along a given line to evaluate the wear performance of the coating. Figure 8 shows the depth and width profiles of wear lines formed during RT dry sliding tests. These profiles are obtained along the lines denoted in the 3D projections given in Figure 7. The depths of these profiles indicate that all ceramic coatings are removed from the surfaces by the test; however, the resistance of the individual coatings to the wear conditions varies. The AlTiN-coated surface after heat treatment shows a shallower profile compared to the CrN-coated surface due to its higher hardness and better adhesion to the substrate [37]. On the other hand, the depth and width of the profile become flatter for the CrN/AlTiN coating, which has the highest hardness in this group of coatings (Figure 8a). For the nitrided and coated surfaces, shallower profiles are formed during tribological interaction, and due to their higher surface hardness, both the depth and width of these profiles decrease (Figure 8b). This result can be attributed to the increase in load-carrying capacity due to the gradual increase in hardness as a result of nitriding [23,42]. Volume loss values after RT wear tests are determined for each coating by using wear profiles of these surfaces. Attained values are divided by the nominal load and sliding distance to determine the specific wear rates (W). Although this approach, suggested by Holm and Archard [35], cannot be considered as a universal wear formula, it reveals the inverse relationship between hardness and volume loss and provides information for comparing the wear performance of surfaces. On the other hand, it is known that the elastic modulus of a coating relative to the that of the substrate has a stronger effect on the potential for yielding in both the coating and the substrate than on the hardness [43]. Wear rates decrease as surface hardness increases, and further decreases in wear rate can be achieved by nitriding (Table 4). Thus, the lowest wear rate of 1.80 × 10^−^^6^ mm^3^/Nm is observed for the CrN/AlTiN coating after nitriding. The higher modulus of elasticity of the AlTiN coating compared to CrN is therefore reflected in its higher load-carrying capacity, as it has a higher stiffness on the same substrate. Functionally graded design in terms of elastic modulus improves the load-carrying capacity in the CrN/AlTiN coating, since the stresses formed during tribological interaction are transferred to the upper layer, which has the highest elastic modulus in the integral system [37,44]. This improvement in load-carrying capacity is even more clear for the nitrided and integral coated surfaces (Table 4).

**Figure 9:**
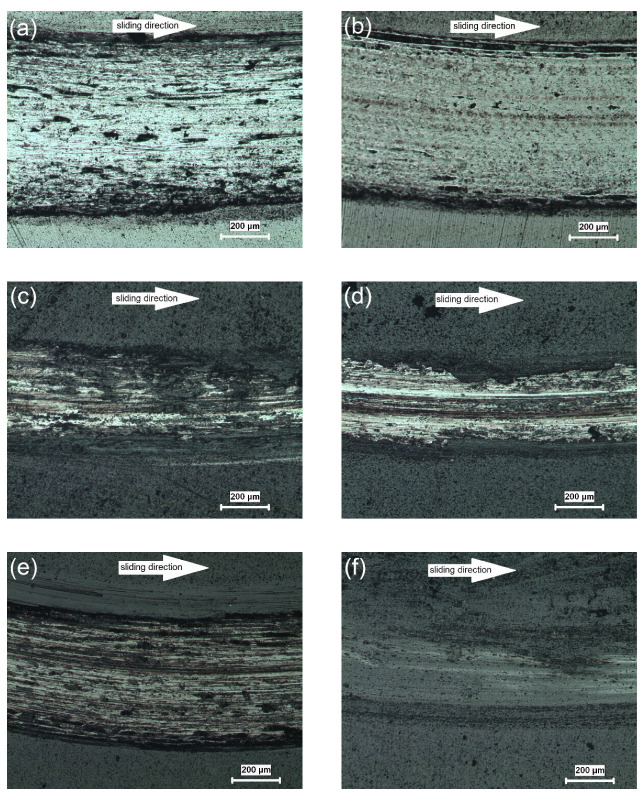
LM images of the worn surfaces of the coated steels: (**a**) HC, (**b**) NC, (**c**) HA, (**d**) NA, (**e**) HCA, and (**f**) NCA.

**Figure 10:**
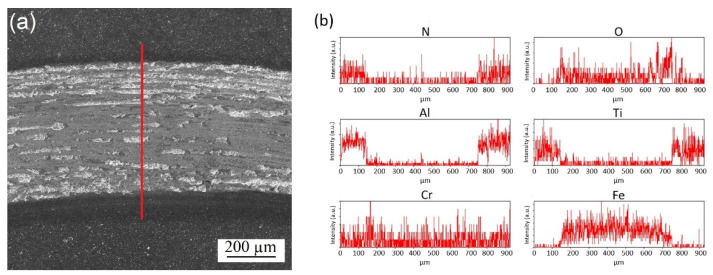
SEM image of the worn surface of (**a**) HCA and (**b**) corresponding line spectra.

**Figure 11:**
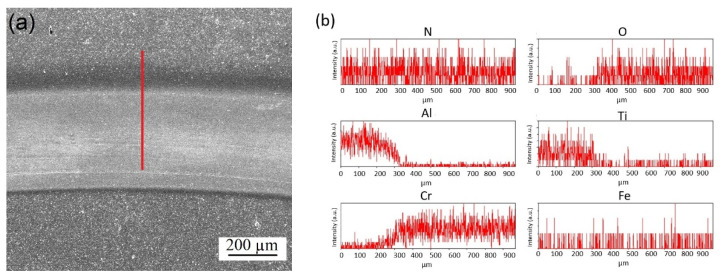
SEM image of the worn surface of (**a**) NCA and corresponding (**b**) line spectra.

Figure 9 shows optical microscopy (LM) images of the worn surfaces of the coated tool steels after dry sliding tests. On all surfaces, scratches with specific widths are observed, formed along the direction of ball motion. The widths of these scratches correlate with the profiles determined by the 3D projections. There are many abrasive scratches parallel to each other and some deep elongated grooves on the surface of the heat-treated and CrN-coated steel (Figure 9a). After nitriding, these characteristic features are observed less frequently, and the surface appears smoother (Figure 9b). In the tribological interaction of the alumina–AlTiN pair, a different mechanism with thin abrasive scratches instead of deep grooves is observed, and adhesive layers are present that are cold-welded on the surface. The wear mechanism in this case is mostly adhesive (Figure 9c). In the nitrided sample, on the other hand, the presence of a hard diffusion layer beneath the coating caused a reduction in material transfer from the steel matrix due to adhesive wear, resulting in abrasive scratches along the wear line (Figure 9d). On the heat-treated and CrN/AlTiN-coated surface, a combination of the above formations (abrasive scratches, grooves, adhesive layers) is observed (Figure 9e). After nitriding, however, bilayer coating displayed mostly abrasive wear along with some delaminated areas. In this case, the tribological interaction leads to a reduction in surface roughness (Figure 9f). After the dry sliding tests, SEM-EDS investigations were carried out on the worn surfaces of HCA and NCA, which have the lowest wear rates in their series, as shown in Figure 10 and Figure 11, respectively. The spectra taken along the marked lines from the surface towards the track show that aluminum and titanium, present in the top layer, decrease in the wear track, while Cr, the other component of the integral coating, is still present in the track with the Fe-rich steel matrix. In the case of HCA, the Cr content within the track is lower than on the surface, indicating the lack of a stable coating. Thus, the Fe amount is relatively high due to the coating removal (Figure 10). These profiles are in agreement with the profilometric measurements. In the nitrided sample, a significant increase in the Cr content is observed. Despite the removal of the top AlTiN layer during the tribological interaction, the other component of the bilayer coating, namely CrN, is wear-resistant and still present on the worn surface (Figure 11). This shows that the gradual increase in hardness from the matrix to the top layer has a functional effect on the wear resistance.

### 3.3. Evaluation of Tribology Test Results at HT

**Figure 12:**
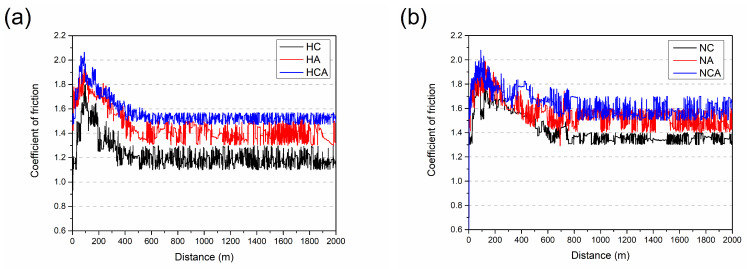
Friction coefficients as a function of distance in HT tribology tests (**a**) coated and (**b**) nitrided-coated specimens.

In Figure 12, the coefficient of friction versus distance graphs for the coatings during tribological interaction with the cylindrical aluminum counterpart material at elevated temperature are given. As observed in earlier studies, there are high fluctuations in the COF values, which are related to the oxidation of aluminum at the test temperature, the occurrence of repeated oxidation during contact, and the adherence and removal of aluminum to and from the surface [32,45,46,47]. There are three stages in these graphs similar to the RT wear tests. The run-in stage is observed within the first 100 m for the heat-treated and coated samples. In this stage, the COF values reach about 1.7, 1.9, and 2.0 for HC, HA, and HCA, respectively (Figure 12a). These high COF values are mainly due to the adherence of aluminum from the counterpart material to the coated surface [32,36,37]. The presence of aluminum in HA and HCA increases the adhesion between Al and Al under the test conditions, causing even higher COF values [37,47]. The nitrided and coated surfaces reveal similar COF values for in the run-in stage within the first 100 m (Figure 12b). On the other hand, the transition stage is between 100m and 500 m for the heat-treated and coated steels (Figure 12a) but ranges between 100 m and 750 m for the nitrided and coated ones (Figure 12b). In this stage, the COF values have a decreasing tendency. Such a decrease in COF values is due to the initiation of plastic deformation of the aluminum in the tribological interaction at high temperatures and to the decrease in Hertzian contact stress. Kalin and Jerina reported for a similar tribological process that (i) the yield strength of the aluminum alloy used as counterpart material decreases significantly with increasing temperature (200 °C and above) and that (ii) the Hertzian contact stresses could decrease by a factor of 30 compared to the value at room temperature due to the reduction of the elasticity limits [32,46]. 

**Figure 13:**
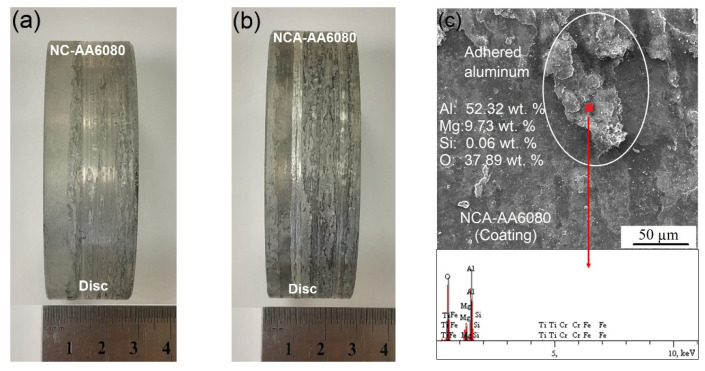
Worn surfaces of the tribological pairs: (**a**) disc surface of NC-AA6080, (**b**) disc surface of NCA-AA6080, and (**c**) coating surface of NCA-AA6080.

**Figure 14:**
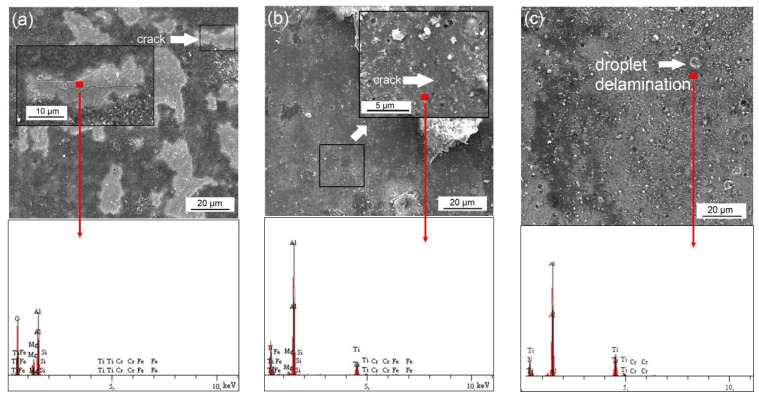
SEM images of the worn surface of the heat-treated and coated steels: (**a**) HC, (**b**) HA, and (**c**) HCA.

**Figure 15:**
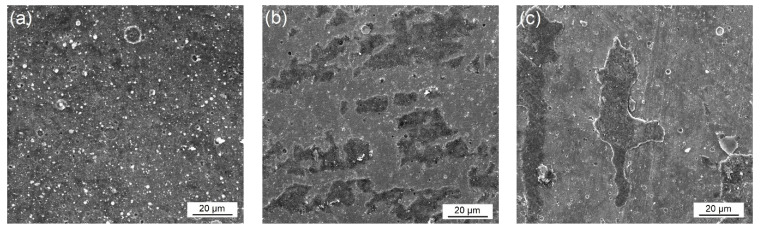
SEM images of the worn surface of the nitrided and coated steels: (**a**) NC, (**b**) NA, and (**c**) NCA.

As can be clearly seen in Figure 12, the COF values have a decreasing tendency until the beginning of the steady state. As a result of the complete removal of surface roughness and the softening of the surfaces at high temperature, a steady wear period begins, and an average COF value is attained in the steady state. In this stage, the COF values are about 1.2, 1.4, and 1.5 for HC, HA, and HCA, respectively. After nitriding and coating, the COF values increase to 1.3, 1.5, and 1.6 for NC, NA, and NCA, respectively. A significant Al–Al adhesion takes place in the steady state, causing higher COF values for HA, HCA, NA, and NCA surfaces. The macroscopic images of the worn aluminum discs, used as counterpart materials for the high-temperature wear tests, are given in Figure 13. Due to the stronger aluminum adhesion, the surface of the disc material in the tribological pair AlTiN–AA6080 has more delaminated and adhered areas (Figure 13b) compared to the one in the tribological pair CrN–AA6080 (Figure 13a). SEM-EDS images show that the adhered layer on the coated surface is oxidized aluminum (Figure 13c). Although the adhesive layers were removed from the surfaces by caustic solution before the SEM studies, the images reveal that they are still present on the surfaces to some extent (Figure 14). Therefore, the measurement of weight loss in the samples is ignored. SEM-EDS investigations of the cleaned surfaces contributed to the understanding of the high-temperature wear performance of different coatings as well as of the effect of nitriding prior to the coating process. As can be seen in Figure 14a, typical cracks are observed in the HC coating tested at elevated temperature. The EDS analysis indicates that the crack occurred in the CrN coating (78.52 Cr, 21.48 N, wt.%). Similar cracks are observed for HA as well, although they are much finer and more branched compared to HC (Figure 14b). The EDS analysis of the HA surface tested at elevated temperature reveals that the coating (42.03 Al, 28.58 Ti, 29.39 N, wt.%) is still present on the tool steel. The numerous SEM analyses of the integral coating (HCA) did not reveal any cracks. On the other hand, droplet delamination and a stable ceramic film structure are present on the HCA surface (Figure 14c), as shown by the EDS analysis (42.86 Al, 28.10 Ti, 5.79 Cr, 23.25 N, wt.%). As already mentioned, the load-carrying capacity of the substrate increases, higher hardness values are achieved compared to heat-treated and coated substrates, and the plastic behavior of the substrate decreases due to the diffusion layer formed by nitriding. SEM studies of worn NC, NA, and NCA surfaces with well-adhered coatings in expanded areas on the substrate demonstrate their higher wear resistance under high-temperature conditions, which is due to a combination of these effects (Figure 15). There are randomly distributed fine scratches as well as droplet delamination on the nitrided and coated steels. This type of delamination is clearly visible on the worn surface of NC (Figure 15a) but is comparatively less so on those of NA and NCA, which have stronger aluminum adhesion (Figure 15b,c).

## 4. Conclusions

In this study, heat-treated and nitrided DIN 1.2367 tool steel coated with CrN, AlTiN, and CrN/AlTiN was subjected to room and elevated temperature wear tests. Therefore, a diffusion layer (~100 µm) was formed on the surface of conventional heat-treated DIN 1.2367 steel (538 HV_10_) by gas nitriding to achieve a hierarchical hardness level before CAPVD coating. As a result of the presence of hard nitrides embedded within the steel matrix, a hardness depth profile ranging between 800 and 538 HV_10_ was provided. Homogeneous and continuous hard CrN (2032 HV_0.01_), AlTiN (2234 HV_0.01_), and CrN/AlTiN (2298 HV_0.01_) coatings were formed on both nonnitrided and nitrided steel surfaces. Room temperature wear tests showed that CrN/AlTiN-coated steel has the highest coefficient of friction value and the lowest specific wear rate among the multisurface engineered materials, and the wear rates of the nitrided and coated steels were further reduced in the interaction with the alumina ball. In order to simulate the aluminum extrusion conditions, the hot wear behavior of the surfaces against AA6080 alloy at 450 °C was also investigated. The hot wear results revealed that (i) the adhesive characteristic of aluminum to the surfaces is responsible for the high friction coefficients, (ii) the nitrided and CrN/AlTiN-coated sample exhibited the lowest wear rate among all studied surfaces, and (iii) drop delamination as a well-known film damage was frequently observed on the tested surfaces.

## Figures and Tables

**Table 1:** The coating parameters applied on the steels.

Coating Type	Cathode Arc Current (A)	Bias Voltage (V)	Coating Time (min)	Nitrogen Partial Pressure (mTorr)
CrN	60	110	70	6.5
AlTiN	50	200	30	8
CrN/AlTiN	80/60	120/100	60/60	6.5/7.0

**Table 2:** Codes and conditions for the studied steels.

Designation	Condition	Designation	Condition
HC	Heat treated + CrN coated	NC	Nitrided + CrN coated
HA	Heat treated + AlTiN coated	NA	Nitrided + AlTiN coated
HCA	Heat treated + CrN/AlTiN coated	NCA	Nitrided + CrN/AlTiN coated

**Table 3:** Surface hardness of the studied steels.

Designation	Hardness (HV_0.01_)	Designation	Hardness (HV_0.01_)
HC	1670 ± 85	NC	2010 ± 92
HA	1869 ± 90	NA	2446 ± 101
HCA	1987 ± 87	NCA	2692 ± 108

**Table 4:** Wear rates for RT tests of the studied steels.

Designation	Wear Rate (×10^−6^ mm^3^/N·m)	Designation	Wear Rate (×10^−6^ mm^3^/N·m)
HC	11.20	NC	9.57
HA	7.76	NA	7.36
HCA	4.30	NCA	1.80

## Data Availability

The data presented in this study are available on request from the corresponding author.
